# Nanoparticle Targeting and Cholesterol Flux Through Scavenger Receptor Type B-1 Inhibits Cellular Exosome Uptake

**DOI:** 10.1038/srep15724

**Published:** 2015-10-29

**Authors:** Michael P. Plebanek, R. Kannan Mutharasan, Olga Volpert, Alexandre Matov, Jesse C. Gatlin, C. Shad Thaxton

**Affiliations:** 1 Northwestern University, Feinberg School of Medicine, Department of Urology, Tarry 16-703, 303 E. Chicago Ave., Chicago, IL 60611 United States; 2 Simpson Querrey Institute for BioNanotechnology, 303 E. Superior St., Chicago, IL 60611 United States; 3 Feinberg Cardiovascular Research Institute, 303 E. Chicago Ave., Tarry 14-725, Chicago, IL 60611 United States; 4 University of Wyoming, Department of Molecular Biology, 1000 E. University Ave., Laramie, WY 82071 United States; 5 University of California at San Francisco, Department of Cell and Tissue Biology, San Francisco, CA 94143 United States; 6 International Institute for Nanotechnology (IIN), Northwestern University, 2145 Sheridan Rd., Evanston, IL 60208 United States

## Abstract

Exosomes are nanoscale vesicles that mediate intercellular communication. Cellular exosome uptake mechanisms are not well defined partly due to the lack of specific inhibitors of this complex cellular process. Exosome uptake depends on cholesterol-rich membrane microdomains called lipid rafts, and can be blocked by non-specific depletion of plasma membrane cholesterol. Scavenger receptor type B-1 (SR-B1), found in lipid rafts, is a receptor for cholesterol-rich high-density lipoproteins (HDL). We hypothesized that a synthetic nanoparticle mimic of HDL (HDL NP) that binds SR-B1 and removes cholesterol through this receptor would inhibit cellular exosome uptake. In cell models, our data show that HDL NPs bind SR-B1, activate cholesterol efflux, and attenuate the influx of esterified cholesterol. As a result, HDL NP treatment results in decreased dynamics and clustering of SR-B1 contained in lipid rafts and potently inhibits cellular exosome uptake. Thus, SR-B1 and targeted HDL NPs provide a fundamental advance in studying cholesterol-dependent cellular uptake mechanisms.

Exosomes transport molecular cargo to and from cells as a means of intercellular communication^1^,^2^, and play a fundamental role in biology^3^. For example, exosomes isolated from stem cells have been shown to increase tissue regeneration after injury^4^,^5^. Additionally, exosomes play an important role in the immune system, through the delivery of major histocompatibility complexes (MHCs)^6^,^7^. Exosomes also contribute to many diseases^1^,^8^,^9^, including cancer^10^,^11^. Cancer cells enhance their production of exosomes as a means of facilitating disease progression^12^,^13^. For example, exosomes produced by melanoma cells have been shown to target endothelial cells to enhance angiogenesis^14^, as well as macrophages and dendritic cells causing immune suppression^15^. In addition, considerable data are accumulating showing that enhanced exosome production by cancer cells facilitates metastasis by conditioning the pre-metastatic niche through the mobilization of bone marrow cells^16^ and the delivery of pro-tumorigenic cargo to metastatic sites^11^.

Specific receptors on target cells that exosomes utilize for uptake are not well known^17^. Data show that target cells uptake exosomes by directly fusing with the plasma membrane^18^, as well as via receptor mediated endocytosis^19^. Because exosome-cell interactions are believed to be critical events to information transfer between the exosome and the target cell, further understanding fundamental mechanisms of these interactions may open avenues for studying intercellular communication and lead to new therapies^19^. Key to this effort is the identification of specifically targeted agents that potently inhibit cellular exosome uptake^19^. Recent data show that exosome uptake by target cells is dependent upon the integrity of plasma membrane microdomains known as lipid rafts, which are known to be rich in cholesterol^20^. Non-specific depletion of plasma membrane cholesterol alters lipid raft integrity and inhibits cellular exosome uptake^21^.

Scavenger receptor type B-1 (SR-B1) is a high-affinity receptor for mature high-density lipoproteins (HDL) that are rich in cholesterol and cholesteryl ester. Upon binding SR-B1, HDL mediates the bi-directional flux of free cholesterol between the HDL particle and the plasma membrane, and serves as a source of cholesteryl ester^22^,^23^. Scavenger receptor type B-1 resides in plasma membrane lipid rafts^24^ where it maintains cholesterol balance and enables the uptake of extracellular material and cell signaling^25^. Our group developed a synthetic, functional HDL-like nanoparticle (HDL NP)^26^,^27^,^28^ that binds SR-B1^26^,^29^. HDL NPs are synthesized using a gold nanoparticle (AuNP) as a core template, and then decorated with the surface molecules, phospholipids and apolipoprotein A-I (apo AI), consistent with the surface chemistry of natural, mature spherical HDLs^26^. HDL NPs are highly functional with regard to their ability to bind SR-B1 and efflux free cholesterol^26^. Because of the core AuNP, HDL NPs are inherently devoid of cholesteryl ester. As such, HDL NPs bind SR-B1 and differentially modulate cellular cholesterol homeostasis relative to their cholesterol-rich natural HDL counterparts^26^,^29^.

Due to the localization of SR-B1 to lipid rafts and the dependence of exosome uptake on cholesterol balance in the plasma membrane, we hypothesized that specific targeting of SR-B1 with cholesterol binding HDL NPs^26^,^27^,^28^,^29^ would disrupt cellular exosome uptake. As a model, we explored exosomes derived from cultured melanoma cells due to the established importance of the uptake of these exosomes by melanoma and other target cells^11^,^15^,^30^,^31^, and because melanoma exosomes have been shown to promote disease progression whereby targeted inhibitors of this process may be translationally relevant^30^,^32^.

## Results

### Exosome Isolation and Characterization

To isolate melanoma-derived exosomes, A375 melanoma cells were cultured and exosomes released into the media were isolated using differential ultracentrifugation^33^. Transmission electron microscopy (TEM) and dynamic light scattering (DLS) measurements indicated vesicular structures of the expected morphology and size (30–100 nm) for exosomes, respectively (Supplementary Fig. 1a,b). Western blot was used to determine the presence of exosome-specific protein cargo further confirming the identity of isolated structures as exosomes (Supplementary Fig. 1c). Interestingly, we found that A375 cells express SR-B1 and exosomes from this cell line are also enriched for the receptor (Supplementary Fig. 1c). These results demonstrate the successful isolation of melanoma-derived exosomes for experiments.

### HDL NPs modulate cholesterol flux in melanoma cells

**Figure 1:**
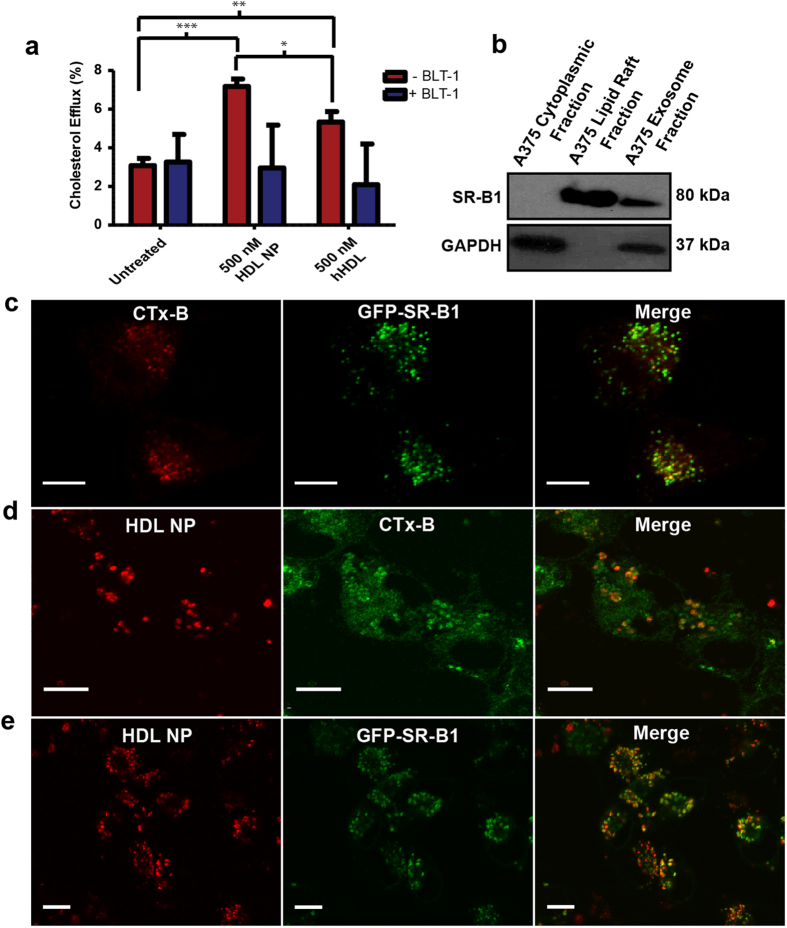
HDL NPs efflux cholesterol and specifically target SR-B1 in melanoma cells. (**a**) ^3^H-cholesterol efflux from A375 cells to HDL NPs (500 nm, final) or hHDL (500 nm, final) was measured with and without BLT-1 treatment (1μM). (**b**) Cells were fractionated using Focus™ Global Fractionation (G Biosciences). Western blot shows SR-B1 enrichment in lipid rafts, presence in exosomes, and absence in the cytoplasmic cell fraction. (**c-e**) Confocal fluorescence microscopy of A375 melanoma cells (live) to assess co-localization of lipid rafts, HDL NPs, and GFP-SR-B1. (Scale bar = 10 μM) (**c**) A375 cells expressing a GFP-SR-B1 fusion protein (green) are stained with an Alexa Fluor-647 conjugated CTx-B (red) to label and image lipid rafts. (**d**) A375 melanoma cell lipid rafts were stained with an Alexafluor-488 conjugated CTx-B (green) after treatment with 20 nm DiD-labeled HDL NPs (red). (**e**) A375 melanoma cells expressing a GFP-SR-B1 fusion protein (green) were treated with DiD labeled HDL NPs (20 nm, red).

High-density lipoproteins are dynamic natural nanostructures that function to sequester, transport, and deliver cholesterol^34^. Many of the physical properties and functions of natural HDLs can be mimicked by HDL NPs^26^, which are synthesized using a 5 nm diameter core AuNP template. The template controls final conjugate size and shape and provides a surface for the assembly of apo AI and phospholipids^28^. Comparison of HDL NPs to certain spherical human HDL (hHDL) species reveals similarities with regard to size, shape, surface chemistry, and negative surface charge^26^,^27^,^35^. Functionally, hHDLs bind SR-B1 and mediate the bi-directional flux of free cholesterol between the particle and the plasma membrane and transfer esterified cholesterol, found in the particle core, to the recipient cell^25^. HDL NPs have been shown to mediate bi-directional free cholesterol flux through SR-B1, like hHDL^27^,^28^; however, the AuNP core of HDL NPs occupies the same physical space as esterified cholesterol does in spherical hHDL rendering HDL NPs incapable of delivering to cells a similar payload of cholesteryl ester^26^,^29^. To clearly demonstrate this, we measured free and esterified cholesterol contained in hHDL and HDL NPs. Data reveal a lack of both free and esterified cholesterol in freshly synthesized HDL NPs (Supplementary Fig. 2), as expected. hHDLs were found to have ~19% free and ~81% esterified cholesterol (percent of total measured cholesterol; Supplementary Fig. 2). Based on these results, we predicted that hHDLs and HDL NPs would exhibit differential effects on cholesterol flux in the A375 melanoma cells. To test this, we labeled the cellular cholesterol pool in melanoma cells using ^3^H-cholesterol, and then performed cholesterol efflux assays to measure the removal of ^3^H-cholesterol from these cells. Data show that HDL NPs induce cholesterol efflux at higher levels than hHDLs (Fig. 1a). Treatment of cells with Blocks Lipid Transport 1 (BLT-1), an inhibitor of SR-B1-mediated cholesterol flux^36^, resulted in reduced efflux to both hHDLs and HDL NPs (Fig. 1a) suggesting that cholesterol efflux is, at least in part, mediated by specific targeting of the SR-B1 receptor by hHDLs and HDL NPs. After the efflux assay, hHDLs and HDL NPs were measured to have increased free cholesterol (percent of total measured cholesterol); however, there still was no measurable esterified cholesterol in HDL NPs versus hHDLs (Supplementary Fig. 2). Cell viability assays demonstrate that despite the increased cholesterol efflux induced by HDL NPs, treatment with HDL NPs at 50 and 100 nm doses does not result in reduced A375 cell viability (Supplementary Fig. 3) at time points up to 72 hours. Thus, cholesterol and cholesteryl ester-poor HDL NPs are not inherently toxic to A375 melanoma cells, target SR-B1, and differentially modulate cholesterol flux through this receptor. These functionally distinct properties of HDL NPs prompted us to probe biological processes, like exosome uptake, that are dependent upon cholesterol.

### HDL NPs localize to scavenger receptor type-B1, which resides in lipid rafts

The mechanistic link between lipid raft integrity and the role that these cell membrane microdomains play in exosome uptake^21^ led us to test whether SR-B1 and HDL NPs localize to lipid rafts in melanoma cells. Consistent with published results^24^, analysis of lipid raft associated proteins via western blot confirmed that SR-B1 localizes to lipid rafts in A375 melanoma cells and showed that SR-B1 is enriched in the insoluble lipid raft membrane fraction compared to the cytoplasmic fraction (Fig. 1b). In complementary experiments, confocal fluorescence microscopy was used to visualize lipid rafts in A375 melanoma cells by labeling the rafts with cholera toxin subunit b (CTx-B) conjugated to Alexafluor-647. We visualized SR-B1 by stably expressing a green fluorescent protein-SR-B1 (GFP-SR-B1) fusion protein in the A375 cells^37^. Expression of the fusion protein was confirmed by western blotting (Supplementary Fig. 4). Imaging revealed co-localization of GFP-SR-B1 with lipid rafts (Fig. 1c). These data establish that lipid rafts in our model melanoma cell line are enriched in SR-B1. To determine whether HDL NPs are targeted to lipid rafts and SR-B1, we treated cells with HDL NPs labeled with a lipophilic fluorescent dye, 1,1′-dioctadecyl-3,3,3′,3′-tetramethylindodicarbocyanine, 4-chlorobenzenesulfonate salt (DiD), and imaged cells to determine co-localization with lipid rafts and SR-B1. Imaging revealed that labeled HDL NPs (red) co-localize with lipid raft CTx-B, labeled with Alexa Fluor-488 (Fig. 1d), and with GFP-SR-B1 (Fig. 1e).

### HDL NPs induce clustering and reduced mobility of SR-B1

**Figure 2:**
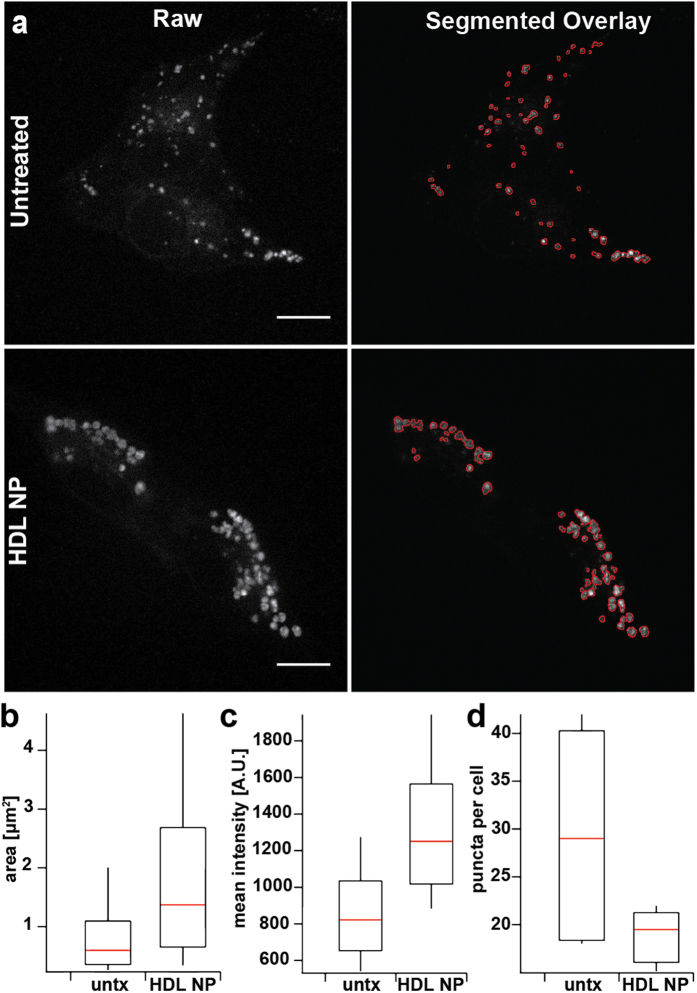
HDL NPs induce clustering of scavenger receptor. Time-lapse images of A375 melanoma cells expressing GFP-SR-B1 were taken in the presence (HDL NP) and absence (untreated, untx) of HDL NPs (30 nm) 24 hours after treatment. (**a**) Representative confocal images of GFP-SR-B1 expressing cells under indicated experimental conditions. Raw images (left) were segmented using a wavelet-based method (see Materials and Methods) to define and measure GFP-SR-B1-positive domains. Outlines of detected clusters are superimposed over the original raw to demonstrate the robustness of segmentation approach used for automatic detection and tracking of the GFP-SR-B1 containing domains (right; scale bar = 10 μM). For each condition, six time-lapse movies (2 minute duration, 2 s lapse) were acquired with n ≥ 15 cells/condition. (**b**) The distribution of areas for all domains present in the first image of each series (red dots; *p ≤ 0.05 via permutation t-test) presented as box plots. Median, the 25th and 75th percentile are shown. Whiskers extend between the 10th and the 90th percentile. (**c**) Average domain brightness per domain: increased brightness in the presence of HDL NPs suggests elevated SR-B1 concentration per area (*p < 0.05 via permutation t-test). (**d**) Average number of GFP-SR-B1 domains per cell for the indicated conditions. Note significantly reduced number of GFP-SR-B1 containing domains per cell as upon HDL NP treatment. (***P < 0.00005 via permutation t-test).

**Figure 3:**
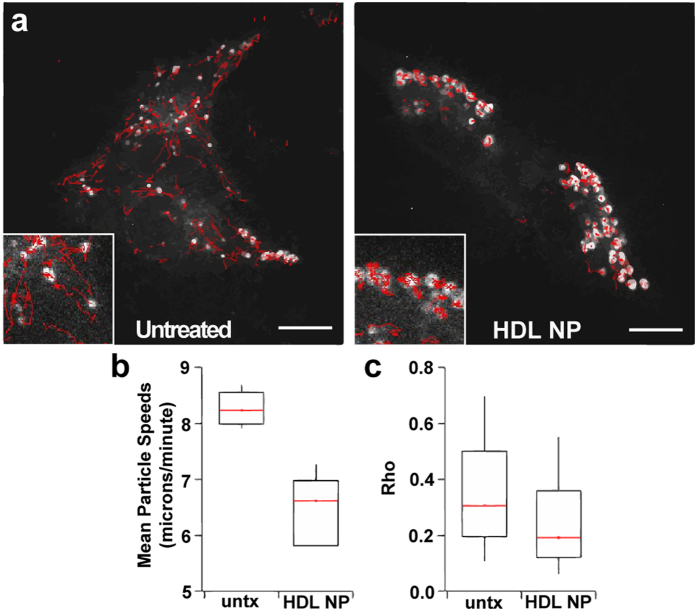
HDL NPs lead to reduced mobility and dispersion of SR-B1 containing domains. Time-lapse confocal imaging (2s intervals) was used to visualize the dynamics of SR-B1 containing domains. Individual domains were detected and tracked as described (see Materials and Methods). (**a**) Motion tracks from the entire duration of imaging overlaid on a single snapshot from the series (untreated cells, left, or HDL NP treatment, right). Insets provide higher magnification images of selected areas with multiple tracks (scale bar = 10 μM). (**b**) Average speeds per puncta for each condition (***P < 0.00005 via permutation t-test). (**c**) The ratio of net displacement (the straight-line distance from the starting point to the end point) to total track length traveled for each GFP-SR-B1 containing domain (*rho*). Values near 1 indicate directed motion.

During the co-localization experiments, we imaged cells treated with HDL NPs at different time points. Intriguingly, images collected at 24 hours revealed physical clustering of GFP-SR-B1 (Fig. 2a, Supplementary Fig. 5) in a dose dependent manner and time-lapse microscopy revealed an apparent reduction in movement and displacement of the receptor upon the addition of HDL NPs (Supplementary videos 2 and 4) as compared to untreated (Supplementary videos 1 and 3). To quantify these observations, we used automated image analysis (*Materials and Methods*)^38^,^39^. Data confirm an increase in the size and intensity of GFP-SR-B1 clusters, and a reduction in the number of labeled areas per cell after HDL NP treatment (Fig. 2a–d). Also, we observed that GFP-SR-B1 clusters tended to remain at the cell membrane versus GFP-SR-B1 that was not clustered (Supplementary Video 1–4). This prompted us to perform tracking analysis to measure GFP-SR-B1 displacement (Fig. 3a). Data revealed a significant quantitative reduction in the velocity (Fig. 3b) and in the ratio of the final displacement relative to the total displacement length (*rho*) of GFP-SR-B1 clusters (Fig. 3c). Collectively, these data suggest that that HDL NPs bind SR-B1 in lipid rafts leading to clustering and arrested movement of GFP-SR-B1.

### HDL NPs inhibit the cellular uptake of melanoma cell-derived exosomes

**Figure 4:**
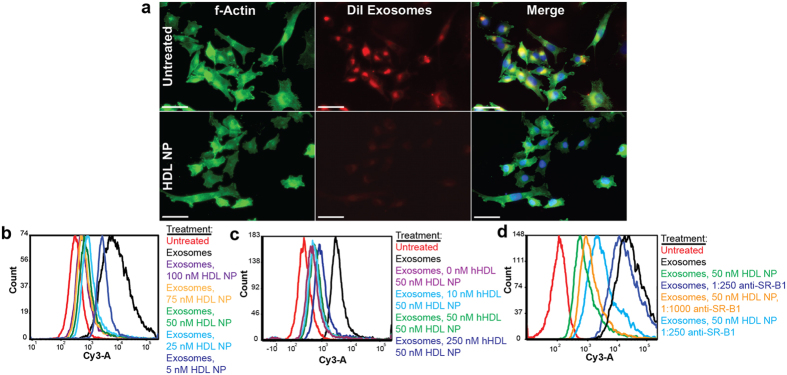
HDL NPs block the uptake of exosomes by A375 melanoma cells. (**a,b**) A375 cells were treated with 1 μg/mL of DiI-labeled exosomes (**a**) Exosome uptake was visualized using fluorescence microscopy after treatment with HDL NP (25 nm, 24 hrs). Actin cytoskeleton was stained using a FITC-phalloidin conjugate and the nuclei were stained with DAPI. The exosome uptake by untreated cells serves as a negative control. (Scale bar = 10 μM) (**b**) DiI-labeled exosome uptake by A375 cells with and without HDL NP treatment (5, 25, 50, 75 and 100 nm HDL NP, 24 hrs) was analyzed using flow cytometry. Cells that were not exposed to DiI labeled exosomes were used as a negative control. (**c**) Partial rescue of exosome uptake by HDL NPs treatment of A375 cells (50 nm) using hHDL treatment (10, 50, 250 nm). (**d**) Dose dependent recovery of exosome uptake in A375 cells treated with HDL NPs (50 nm, 24 hrs) by anti-SR-B1 antibody.

Cellular uptake of exosomes is dependent on lipid raft-mediated endocytosis^21^. As HDL NPs differentially modulate cellular cholesterol homeostasis and physically modulate SR-B1 localized to lipid rafts, we tested the hypothesis that HDL NPs interfere with cellular exosome uptake. Toward this end, we isolated exosomes from A375 melanoma cells and fluorescently labeled them with 1,1′-dioctadecyl-3,3,3′,3′-tetramethylindocarbocyanine perchlorate (DiI). We then treated the A375 cells with labeled exosomes in the presence or absence of HDL NPs and subsequently measured cell uptake. Confocal fluorescent microscopy revealed that HDL NP treatment decreased exosome uptake as compared to untreated control cells at 24 hours (Fig. 4a). In order to quantify exosome uptake in large numbers of cells we employed flow cytometry. Data demonstrated a dose-dependent decrease in exosome uptake after HDL NP treatment (Fig. 4b). At the 50 nm dose, approximately 75% of exosome uptake by the A375 cells was blocked. Notably, the uptake of exosomes was similar in wild-type and GFP-SR-B1 expressing A375 cells, and similar reductions in exosome uptake after HDL NP treatment were observed in both lines (Supplementary Fig. 6a,b). As a control, we treated GFP-SR-B1 expressing A375 cells with exosomes to determine if GFP-SR-B1 clustering was observed. Data reveal that exosome treatment alone did not result in the clustering of GFP-SR-B1 (Supplementary Video 5) suggesting that this cellular phenotype resulted from HDL NP treatment. Additionally, to test if HDL NPs interact with exosome or A375 cell-associated SR-B1, cells were pre-treated with HDL NPs for 12 hours, washed free of unbound HDL NP, and then treated with DiI labeled exosomes. Reduced exosome uptake following HDL NP pre-treatment suggests that decreased uptake is not due to extracellular interaction of exosomes and HDL NPs (Supplementary Fig. 7a,b).

In our cholesterol flux experiments (Fig. 1b and Supplementary Fig. 2), HDL NP and hHDL both bind to SR-B1, promoting cholesterol efflux through this receptor. To determine whether hHDL had the same effect as HDL NP on inhibiting the cellular uptake of labeled exosomes, we again used flow cytometry. Intriguingly, data show that hHDL treatment only minimally inhibits cellular exosome uptake (Supplementary Fig. 8a,b) compared to HDL NP treatment. Both hHDL and HDL NPs target SR-B1, but only the HDL NPs inhibit exosome uptake, which provided an opportunity to demonstrate that hHDL and HDL NPs compete for the same cell surface receptors involved in exosome uptake. Co-treatment of cells with HDL NP and increasing amounts of hHDL resulted in a partial concentration-dependent recovery in exosome uptake (Fig. 4c) suggesting competition for SR-B1. Based on our observing only a partial recovery, we reasoned that hHDL might also reduce cellular exosome uptake. To test this, A375 cells were co-treated with fluorescently labeled exosomes and hHDL at 5, 50 or 500 nm concentrations and exosome uptake was measured using flow cytometry. hHDL was unable to potently block the uptake of exosomes even at a concentration of 500 nm, which is 10-times the HDL NP concentration required for near complete inhibition of exosome uptake (Supplementary Fig. 8). Accordingly, hHDL does not reduce cellular exosome uptake and the high concentration of hHDL needed to abrogate HDL NP-mediated inhibition of exosome uptake suggests that HDL NPs have a higher binding affinity to cell-surface SR-B1 receptors. Also, the ability of HDL NP to inhibit exosome uptake in comparison to hHDL suggests that binding SR-B1 and differential modulation of cholesterol are mechanistically important in inhibiting exosome uptake.

To more directly test if the HDL NPs specifically target SR-B1 to inhibit exosome uptake, we treated cells with HDL NPs and a blocking antibody (Ab) to SR-B1, which has been shown to inhibit hHDL binding to this receptor^40^. Treatment of A375 cells with the blocking Ab resulted in a significant reduction in the ability of HDL NPs to inhibit exosome uptake (Fig. 4d). Thus, these data support the conclusion that HDL NPs specifically block exosome uptake in melanoma cells by binding SR-B1.

### Targeting SR-B1 to block cellular exosome uptake

**Figure 5:**
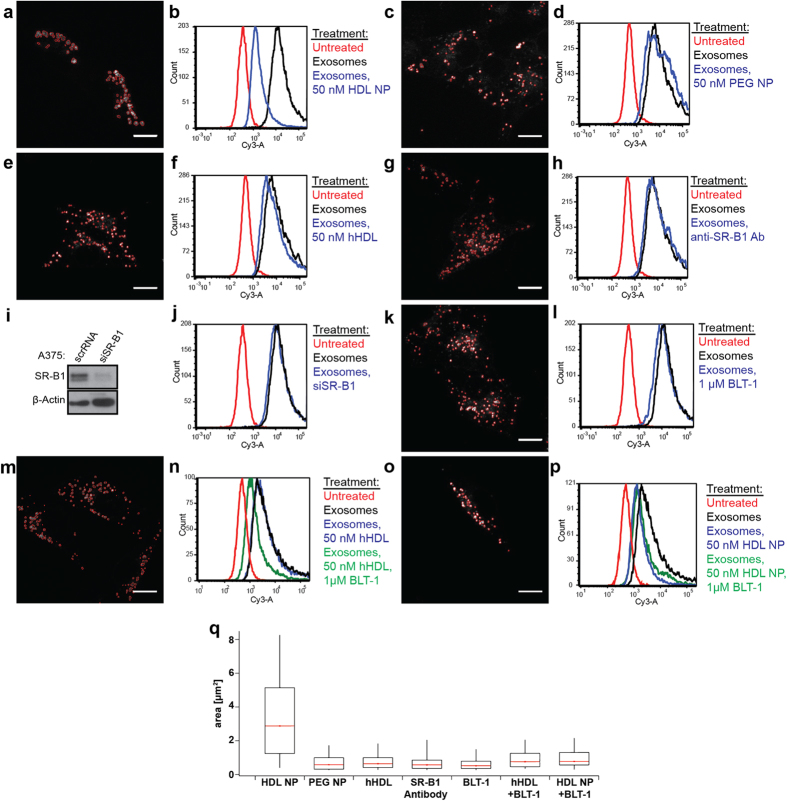
Targeting SR-B1 to induce receptor clustering and inhibit exosome uptake. A375 melanoma cells were analyzed for exosome uptake by flow cytometry and clustering of GFP-SR-B1 containing domains was measured using fluorescent microscopy after 24 hrs treatment with the following agents: (**a,b**) 50 nm HDL NPs; (**c,d**) 50 nm PEG-NPs; (**e,f**) 50 nm hHDL; (**g,h**) SR-B1 neutralizing antibody; (**i,j**) siRNA targeting SR-B1 expression (siSR-B1); and, (**k,l**) 1 μM BLT-1. (**i**) Western blot to confirm SR-B1 knockdown. (**m,n**) Combined treatment of A375 cells with hHDL (50 nm) and BLT-1 (1 μM). (**o,p**) Combined treatment of A375 cells with HDL NP (50 nm) and BLT-1 (1 μM). (**q**) The box plot shows average size of the GFP-SR-B1 positive clusters per experimental condition. Representative fluorescence images are shown for each condition. (Scale bar = 10 μM).

The pronounced effects of HDL NPs on both SR-B1 dynamics and exosome uptake led us to examine the functional importance of individual features specific to the HDL NPs. Structurally, HDL NPs comprise a 5 nm diameter gold core and have the size, shape, and surface chemistry consistent with some hHDL species^27^, but the inherent flexibility of NP synthesis techniques enabled generation of particles with different surface chemistry. This allowed us to measure exosome uptake and SR-B1 clustering after treating A375 cells with particles having an identical gold core, but with passive surface chemistry (polyethylene glycol nanoparticles, PEG NPs). In addition, we also probed individual, functional properties of the HDL NPs by testing if the blocking Ab targeting SR-B1^40^; the small molecule inhibitor of free and esterified cholesterol flux through SR-B1, BLT-1^36^; siRNA targeting melanoma cell SR-B1 expression; or combining HDL NPs and hHDL with BLT-1 would modulate cellular exosome uptake. As measured using flow cytometry, HDL NPs were the only particle or SR-B1 targeted treatment capable of clustering GFP-SR-B1 and inducing potent inhibition of cellular exosome uptake (Fig. 5a-l). These results suggest that HDL NPs occupy SR-B1 and modulate free and esterified cholesterol flux, and that this combination of events results in the clustering of SR-B1 and the disruption of cellular exosome uptake. Finally, our data suggest that cellular exosome uptake is, at least in part, not responsive to a reduction in cellular SR-B1 expression (Fig. 5i,j); however, specific binding of this receptor by HDL NPs is a potent, targeted mechanism to inhibit cellular exosome uptake.

To more conclusively support the mechanism of action of the HDL NPs, we co-treated A375 melanoma cells with HDL NPs or hHDL and BLT-1 to simultaneously occupy SR-B1 and block free and esterified cholesterol flux through the receptor, respectively. Intriguingly, data show that combining either hHDL or HDL NPs with BLT-1 potently inhibits exosome uptake (Fig. 5n,p). However, while some clustering of SR-B1 is observed, data show that there is less than when HDL NPs are used as a single agent (Fig. 5m,o,q) Therefore, as the only functional difference between treating cells with HDL NP alone versus in combination with BLT-1 is the particle’s ability to support free cholesterol flux, this function of the HDL NP is critical to clustering SR-B1. On the other hand, because exosome uptake is potently inhibited after combining HDL NP and hHDL with BLT-1, and since there is only a very slight increase in exosome uptake in the HDL NP + BLT-1 case, data support that the inhibition of exosome uptake can be attributed to SR-B1 binding and inhibition of cholesteryl ester influx.

### Exosome uptake is not inhibited by HDL NPs after SR-B1 knockdown in A375 cells

**Figure 6:**
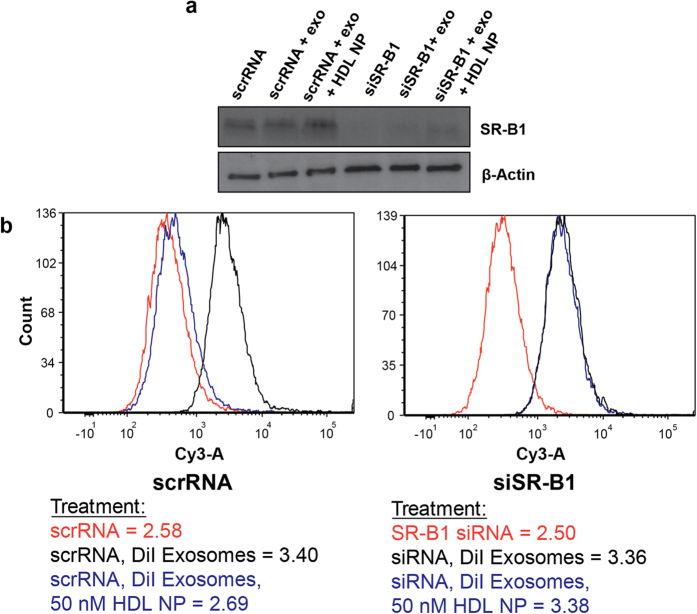
Expression of SR-B1 is required for HDL NP-mediated inhibition of exosome uptake. Wild-type A375 melanoma cells were treated with scrambled siRNA or siRNA-targeted to SR-B1. (**a**) Western blot reveals a reduction in SR-B1 expression in A375 cells at 48 hours after transfection with targeted siRNA. No reduction is measured in cells treated with scrambled siRNA. (**b**) Flow cytometry reveals a drastic reduction in labeled exosome uptake in the presence of HDL NPs (50 nm) in the cells treated with scrambled siRNA after 24 hours treatment. However, no significant reduction in labeled exosome uptake is observed upon HDL NP treatment in the case where siRNA expression has been reduced. The mean fluorescent intensity values (log scale) are included next to each histogram.

In order to further show that HDL NPs directly target SR-B1 to inhibit exosome uptake, we reduced SR-B1 expression with SR-B1 targeted siRNA, as in the preceding section, but then treated the cells with labeled exosomes and HDL NPs. Flow cytometry data convincingly show that after SR-B1 knockdown in wild-type A375 cells, confirmed by Western blot (Fig. 6a), exosome uptake is not significantly reduced in the presence of HDL NP treatment (Fig. 6b). Control experiments in wild-type A375 cells treated with scrambled siRNA (Fig. 6b, showing no SR-B1 KD) reveal that HDL NP treatment significantly reduces exosome uptake, as expected. These experiments further and directly implicate HDL NP targeting of SR-B1 as a mechanism to potently reduce cellular exosome uptake.

### The uptake of exosomes by endothelial cells and macrophages is also blocked by HDL NPs

**Figure 7:**
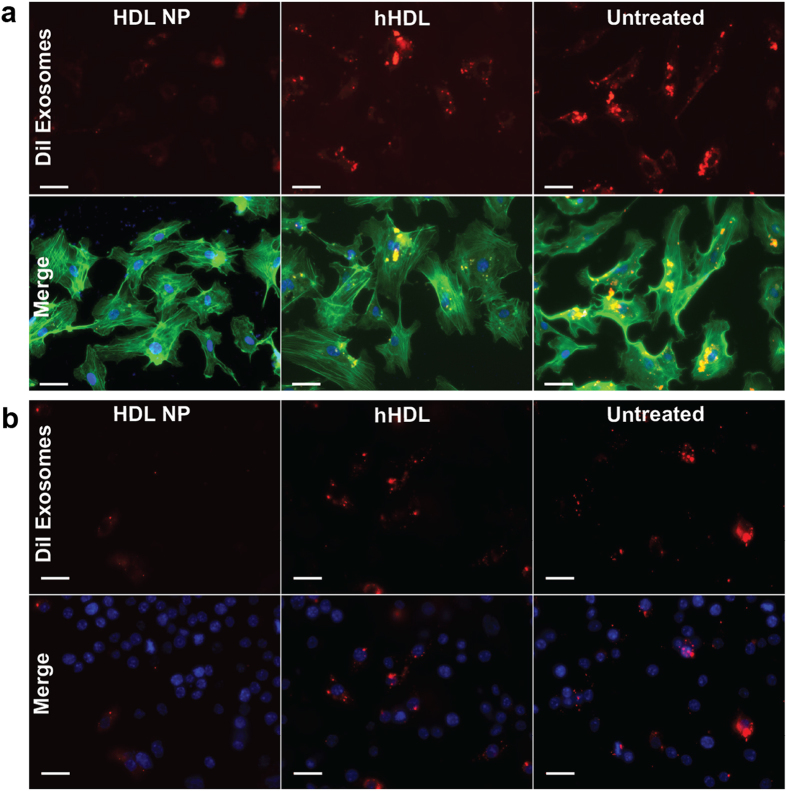
HDL NPs inhibit the uptake of melanoma exosomes by endothelial cells and macrophages. (**a**) HMVECs were treated with 1 μg/mL of DiI labeled exosomes and visualized using fluorescence microscopy after treatment with 25 nm HDL NP or hHDL for 24 hrs. The actin cytoskeleton was stained using a FITC-phalloidin conjugate and the nuclei were stained with DAPI. The exosome uptake of HMVECs after treatment with HDL NPs was compared to hHDL treatment and untreated cells. (**b**) RAW 264.7 macrophages were treated with 1 μg/mL of DiI labeled exosomes and visualized using fluorescence microscopy after treatment with 25 nm HDL NP or hHDL for 4 hrs. (Scale bar: 10 μM).

Data collected using melanoma cells are intriguing, but we were curious if inhibition of exosome uptake by HDL NPs was unique to the A375 melanoma cells or was more general. As mentioned, melanoma exosomes are known to target endothelial cells and macrophages leading to activation of an angiogenic response^31^, and modulation of the immune system^13^. Therefore, we chose two different cell types, an endothelial cell line, human dermal microvascular endothelial cells (HMVECs) and RAW 264.7 macrophages and repeated select experiments to determine SR-B1 expression and HDL NP effect on exosome uptake. Like A375 cells, HMVECs express SR-B1 (Supplementary Fig. 4) and when treated with DiI labeled A375 exosomes in the presence of HDL NPs, these cells exhibited a decrease in cellular fluorescence suggesting that exosome uptake is, indeed, blocked by HDL NP. In contrast, treatment with hHDL had minimal effect on exosome uptake (Fig. 7a). RAW 264.7 macrophages also express SR-B1^41^, so we analyzed exosome uptake in these cells after HDL NP treatment. As was observed with HMVECs, HDL NPs decreased the uptake of exosomes, and hHDL had no effect (Fig. 7b). These *ex vivo* proof-of-concept experiments not only demonstrate that HDL NPs block exosome uptake in cell types shown to be important for melanoma progression, but also suggest that HDL NP may therapeutically modulate intercellular communication events that are critical for melanoma progression.

## Discussion

**Figure 8:**
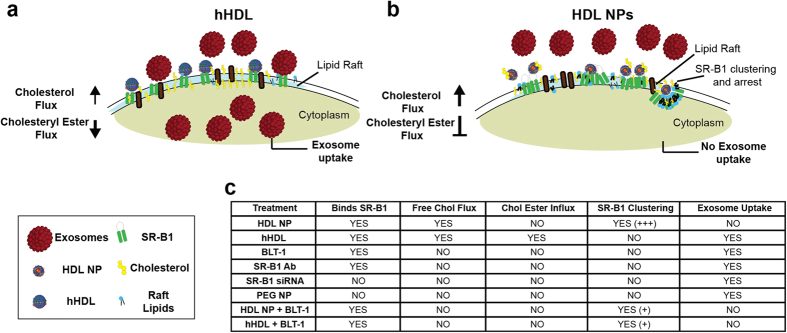
Summary of proposed mechanism by which HDL NPs inhibit exosome uptake: (**a**) In the presence of hHDL cellular exosome uptake is maintained. Through SR-B1, hHDL can remove cellular cholesterol and is a source of esterified cholesterol. (**b**) HDL NPs bind SR-B1 and efflux cholesterol from the cell more efficiently than hHDL (darker arrow) and are not a source of esterified cholesterol (blocked arrow). This leads to SR-B1 receptor clustering and potent reduction in cellular exosome uptake. (**c**) Table that enables easy comparisons to be made between different cell treatments, their function, and the resulting phenotypic clustering and exosome uptake results.

Our data demonstrate that HDL NPs are a targeted and functional nanoconjugate that potently inhibit cellular exosome uptake in cultured melanoma, endothelial, and macrophage cells. Mechanistically, a model (Fig. 8a,b) is proposed for HDL NPs whereby tight binding to SR-B1 in lipid rafts modulates free and esterified cholesterol flux through this receptor. Ultimately, HDL NP binding to SR-B1 and modulating free cholesterol flux is responsible for clustering and stagnating SR-B1 at the cell membrane. Further, HDL NP binding to SR-B1 and blocking the influx of cholesteryl ester leads to a dramatic reduction of cellular exosome uptake. This proposed mechanism is supported by data collected using hHDL and BLT-1 whereby this combination of SR-B1 receptor occupancy and inhibition of cholesterol flux and cholesteryl ester uptake, respectively, potently inhibits cellular exosome uptake. Finally, this mechanism is specific and unique to HDL NPs, and is not shared by another particle that has a gold core and altered, non-HDL-like surface chemistry; by other means of inhibiting SR-B1 using single, targeted agents (i.e. blocking Ab or BLT-1); or by knocking down the cellular expression level of SR-B1 (Fig. 8c).

The combination of HDL NPs and cellular exosome uptake provides a unique nanoparticle and phenotypic output signal that evidently uncouples targeted SR-B1 receptor binding from cholesterol flux to uncover a phenotype that is clearly impacted by local cell membrane cholesterol and cholesteryl ester flux. Further work is required to better understand downstream molecular events that occur upon functional HDL NP binding to SR-B1. Certainly, we appreciate that HDL NPs binding to SR-B1 illicit different responses in specific cell types^29^,^42^, and that cell surface and downstream second messenger signaling events may determine cell phenotype. We anticipate that different cholesterol-dependent phenotypes will be uncovered in other model systems. Finally, we are currently exploring the functional consequences of inhibiting cellular communication events mediated by exosomes in the context of primary and metastatic melanoma models, and beyond.

In summary, our data implicate SR-B1 in the cellular uptake of exosomes in the context of targeted, functional HDL NPs that may prove to be a valuable tool to better understand cholesterol-dependent cellular uptake mechanisms of exosomes and, perhaps more broadly, other extracellular vesicles. Extension of our findings in melanoma cells to endothelial cells and macrophages suggests that the unique mechanism by which exosome uptake is inhibited by HDL NPs may be more general. Further work is focused on elucidating the functional consequences of inhibiting exosome-based information transfer between cells, and identifying cell-signaling pathways that may be altered by HDL NP binding to SR-B1 that may contribute to the observed reduction in cellular exosome uptake.

## Materials and Methods

### HDL NP Synthesis

Biomimetic high-density lipoprotein-like nanoparticles (HDL NPs) were synthesized and characterized as previously described^26^,^27^,^28^,^29^. Briefly, citrate stabilized 5 nm diameter gold nanoparticles (AuNP, Ted Pella) were used as a template for surface chemical modification. Purified human apolipoprotein AI (apoA-I) was incubated with a solution of AuNPs (80 nm) at 5-fold molar excess (400 nm, final) for 1 hour at room temperature (RT) with gentle stirring. Next, the phospholipids, 1-2-dipalmitoyl-sn-glycero-3-phosphocholine and 1,2-dipalmitoyl-sn-glycero-3-phosphoethanolamine-N-[3-(2-pyridyldithio)propionate] were added at 250 molar excess relative to [AuNP] in a mixture of ethanol and water (1:4), and allowed to incubate at RT for 4 hours with gentle stirring. The HDL NPs were then purified and concentrated using tangential flow filtration. The HDL NP concentration and final conjugate size were determined using UV-Vis spectrophotometry (ε_AuNP_ = 9.696 × 10^6^ M^−1^ cm^−1^ at *λ*_max_ = 520 nm) and dynamic light scattering (DLS, Malvern Zetasizer), respectively.

### Cell Culture

A375 melanoma cells (ATCC) and RAW 264.7 macrophages (ATCC) were cultured in Dulbecco’s Modified Eagle Medium (DMEM) containing 10% fetal bovine serum and 1% penicillin/ streptomycin. Human dermal microvascular endothelial cells (HMVECs) and endothelial cell growth medium were from Promocell. Cells were incubated at 37 °C and in a humidified 5% CO_2_ environment. The GFP-SR-B1 plasmid^37^ was stably transfected in the A375 cells using Lipofectamine 2000 (Life Technologies) and transfectants were selected using Geneticin (Life Technologies) followed by fluorescent associated cell sorting (FACS).

### Exosome isolation and labeling

A375 melanoma exosomes were isolated from conditioned media using differential ultracentrifugation^33^. In brief, cells were cultured in exosome deficient media for 72 hours at which point the cell culture media was collected and centrifuged at 2000 × g to remove dead cells and debris. Next, larger vesicles and cell debris were removed by centrifugation at 10,000 × g for 30 minutes. Exosomes were then pelleted by centrifugation at 100,000 × g for 70 minutes, and subsequently washed in PBS by another 100,000 × g centrifugation step for 70 minutes. Exosomes were re-suspended in PBS. Protein concentration of exosomes was analyzed by BCA Protein assay (Thermo Scientific). Exosome size and morphology was characterized using DLS and transmission electron microscopy (FEI Spirit G2 TEM). In the experiments utilizing fluorescently labeled exosomes, the lipophilic dye, DiI or DiD (Life Technologies), was added to the exosome preparation at a concentration of 2.5 μM after the first 100,000 × g ultracentrifugation step. The fluorophore-labeled exosomes were then washed twice in PBS by pelleting the exosomes and discarding the supernatant. Notably, gold nanoparticles demonstrate distance-dependent fluorescence quenching^43^. In order to test if HDL NPs quenched exosome fluorescence, we incubated HDL NPs with fluorescently labeled exosomes for 4 hours and then measured the fluorescent signal. Data demonstrate no reduction in fluorescence indicating that this is not a mechanism of reduced fluorescence in our measurements (Supplementary Fig. 9).

### Cell treatments with hHDL and HDL NP

For cholesterol determination assays, efflux assays, and cell treatments we used equimolar amounts of hHDL and HDL NPs based upon apo A-I concentration. The molar concentration of HDL NP was determined as discussed above, and each HDL NP has approximately three copies of apo A-I^26^,^27^. Therefore, the molar concentration of apo A-I is easily calculated for the HDL NPs. Human HDL was purchased from Calbiochem. The protein concentration of purchased hHDL was provided. From this value, the amount of apo A-I was calculated for hHDL assuming that 70% of the total protein is apo A-I^22^. Thus, for each treatment the amount of apo A-I is equivalent for hHDL and HDL NP and, because each hHDL and HDL NP has approximately three copies of apo A-I^44^, the dose of particles was assumed to be equivalent.

### Exosome uptake assays

The cellular uptake of exosomes was measured by fluorescence microscopy and flow cytometry after cell treatments. A375 cells, HMVECs and RAW 264.7 macrophages were treated with fluorescent exosomes at a concentration of 1 μg/ml (exosomal protein). For fluorescence microscopy experiments, cells were plated on coverslips coated with 0.1% gelatin. Exosome uptake was measured over the course of 24 hours using a BD LSR Fortessa flow cytometer (Robert H. Lurie Comprehensive Cancer Center Flow Cytometry Core) or a Nikon A1R fluorescence microscope (Northwestern University Nikon Imaging Facility).

### Cholesterol and cholesteryl ester quantification

The total cholesterol and cholesteryl ester content of hHDLs and HDL NPs was measured using an Amplex Red cholesterol detection assay (Life Technologies). The free cholesterol content of each sample was measured in the absence and presence of cholesterol esterase to determine the free cholesterol and total cholesterol, respectively. Cholesteryl ester amount was determined by subtracting the free cholesterol from total cholesterol measurement. To determine the free and esterified cholesterol content of hHDL and HDL NPs before cell incubation we followed the protocol supplied by the manufacturer. The free and esterified cholesterol content of the hHDL and HDL NP acceptors was measured after incubating with cultured A375 melanoma cells in serum free media and HDL NP (50 nm, final) or hHDL (50 nm, final) for 24 hours. After the treatment interval, the culture media was collected and centrifuged to rid the media of cells and cell debris. The total cholesterol and free cholesterol was then determined from conditioned media samples using the Amplex Red assay.

### Cholesterol efflux assay

A375 cells were cultured in DMEM containing 1 μCi/mL [1,2,-^3^H] cholesterol (Perkin-Elmer) overnight to label the cellular cholesterol pool. Cells were then washed in PBS and resuspended in serum free media. Human HDL or HDL NPs were added to the culture media and allowed to incubate for 6 hours. Cell culture media was then collected and subjected to liquid scintillation counting. The percentage of cholesterol efflux was determined by using the formula counts media/(counts cells + counts media) × 100. Efflux of cholesterol in the absence of an acceptor was also measured and interpreted along with other results.

### Computer vision analysis of GFP-SR-B1 domains, intensity, and dynamics

A semi-automated approach using ImageJ software was employed to identify the areas of GFP-SR-B1 in the images. After background subtraction, an unsharp mask filter with a large radius was applied to locally enhance contrast. Manual thresholding of filtered images was then used to generate a segment mask, which could be overlaid on the original, background subtracted image to facilitate measurement of domain parameters such as area and mean intensity.

To test differences in the dynamics properties of the GFP-SR-B1 domains, we identified in automated fashion the center of mass of the spots using a wavelet-based segmentation approach^38^ and tracked their displacement^39^. The method used solves a global combinatorial optimization problem whose solution identifies the overall most likely links of particle trajectories throughout a movie. It allows the tracking of the heterogeneous domain motion both during phases of diffusive and linear motion. During the linking part of the algorithm, we allowed speeds of up to 42 microns/min, as we observed some very rapid motion. We did not use the gap closing option of the algorithm, as the fluorescent labeling was consistently bright and the GFP-SR-B1 motion did not result in occlusion. We included in our dynamics analysis tracks with a lifetime of over four frames.

To calculate the linearity of the motion, we introduced a parameter *rho* (Fig. 3c), which is calculated as the ratio between the head-to-tail (first point to the end point) trajectory distance divided by the total distance traveled by fluorescent domains. This way, a trajectory with *rho* close to 1 signifies a linearly moving spot and a trajectory with *rho* close to 0 signifies randomly moving spots. For this analysis, we excluded all stationary areas by considering spots that moved a pixel per frame one average or more.

### Western Blotting

For Western blot, 20 μg of total protein extract or 10 μg of exosomal protein were resolved on Tris/Glycine/SDS pre-cast polyacrylamide gels (a 4–20% gradient, Bio-Rad, 30 minutes at 200 volts). Proteins were transferred onto polyvinylidene fluoride (PVDF) membranes. The membranes were blocked in 5% milk in Tris-buffered saline containing 0.1% Tween 20 (TBST). The membranes were incubated with d primary antibodies (diluted in blocking solution) overnight at 4 °C, was washed 3 times in 0.1% TBST (10 minutes/wash) and incubated with the appropriate HRP-conjugated secondary antibody in blocking buffer for 1 hour at room temperature. The membranes were then washed in 0.1% TBST (3 × 10 min) and developed with ECL kit (GE Healthcare). Antibodies: CD81 and GM130 (Santa Cruz Biotechnology), SR-B1 (Abcam), β-Actin (Cell Signaling Technology)

### Lipid raft labeling

A375 lipid rafts were labeled using cholera toxin subunit b (CTx-B) conjugates with Alexafluor 488 or Alexafluor 647 to (Life Technologies) at a final concentration 1 μg/ml, for 30 minutes at 37 °C^21^. The cells were then washed in PBS. And visualized using fluorescence microscopy.

### Knockdown of SR-B1

SR-B1 was knocked down using siRNA targeted to SR-B1 (Ambion). In brief, SR-B1 siRNA and a non-targeted scrambled control were transfected into cells using RNAi Max (Life Technologies). The RNAi Max and RNA were allowed to incubate with the cells for 16 hrs. Cells were then washed free of the transfection reagent, fresh culture media was added, and then incubated for an additional 24 hours prior to cell treatments with exosomes and HDL NP. SR-B1 knockdown was measured using western blot.

### Fluorescence microscopy

Fluorescence microscopy was performed using an A1R confocal microscope with assistance from the Northwestern University Center for Advanced Microscopy. Images were analyzed using NIS Elements (Nikon) and ImageJ (NIH) software. Live cell confocal fluorescence microscopy to assess lipid raft dynamics was performed with a Nikon Eclipse T1 microscope equipped with an Andor iXon Ultra 897 camera and analyzed using Metamorph software (Molecular Devices).

### Statistical analysis

Data was expressed using ± standard deviation of triplicate experiments. The unpaired two tailed student’s t-test from GraphPad Prism software was used to analyze data. Statistical significance was considered for significant for *P* ≤ 0.05. * Denotes *P* ≤ 0.05, ** *P* ≤ 0.01, and *** *P* ≤ 0.001. FCS Express was used to analyze flow cytometry. Statistical analysis between the conditions (before and after HDL NP treatment) of GFP-SR-B1, integrated normalized intensity, and motion was performed using a permutation test^45^ for means, which does not assume normality of the underlying distributions.

## Additional Information

**How to cite this article**: Plebanek, M. P. *et al.* Nanoparticle Targeting and Cholesterol Flux Through Scavenger Receptor Type B-1 Inhibits Cellular Exosome Uptake. *Sci. Rep.* **5**, 15724; doi: 10.1038/srep15724 (2015).

## Supplementary Material

Supplementary Information

Supplementary Video 1

Supplementary Video 2

Supplementary Video 3

Supplementary Video 4

Supplementary Video 5
