# Supplementary material for: Nanoparticle Targeting and Cholesterol Flux Through Scavenger Receptor Type B-1 Inhibits Cellular Exosome Uptake
Source: Sci Rep. 2015 Oct 29;5:15724. doi: 10.1038/srep15724 (PMC4625174; doi:10.1038/srep15724)
Supplement: Supplementary Information [file srep15724-s1.pdf]

Supplementary Information:

Nanoparticle Targeting and Cholesterol Flux Through Scavenger Receptor Type B-1 Inhibits Cellular Exosome Uptake

Michael P. Plebanek[1,2], R. Kannan Mutharasan[3], Olga Volpert[1], Alexandre Matov[4,5], Jesse C. Gatlin[4], C. Shad Thaxton[1,2,6,*]

[1]Northwestern University, Feinberg School of Medicine, Department of Urology, Tarry 16-703, 303 E. Chicago Ave. Chicago, IL 60611 United States

[2]Simpson Querrey Institute for BioNanotechnology, 303 E. Superior St, Chicago, IL 60611 United States

[3]Feinberg Cardiovascular Research Institute, 303 E. Chicago Ave, Tarry 14-725, Chicago, IL 60611 United States

[4]University of Wyoming, Department of Molecular Biology, 1000 E. University Ave, Laramie, WY, 82071, United States

[5]University of California at San Francisco, Department of Cell and Tissue Biology, San Francisco, CA 94143, United States

[6]International Institute for Nanotechnology (IIN), Northwestern University, 2145 Sheridan Rd. Evanston IL. 60208, United States

*Correspondence: C. Shad Thaxton (cthaxton003@md.northwestern.edu)

Supplementary Material

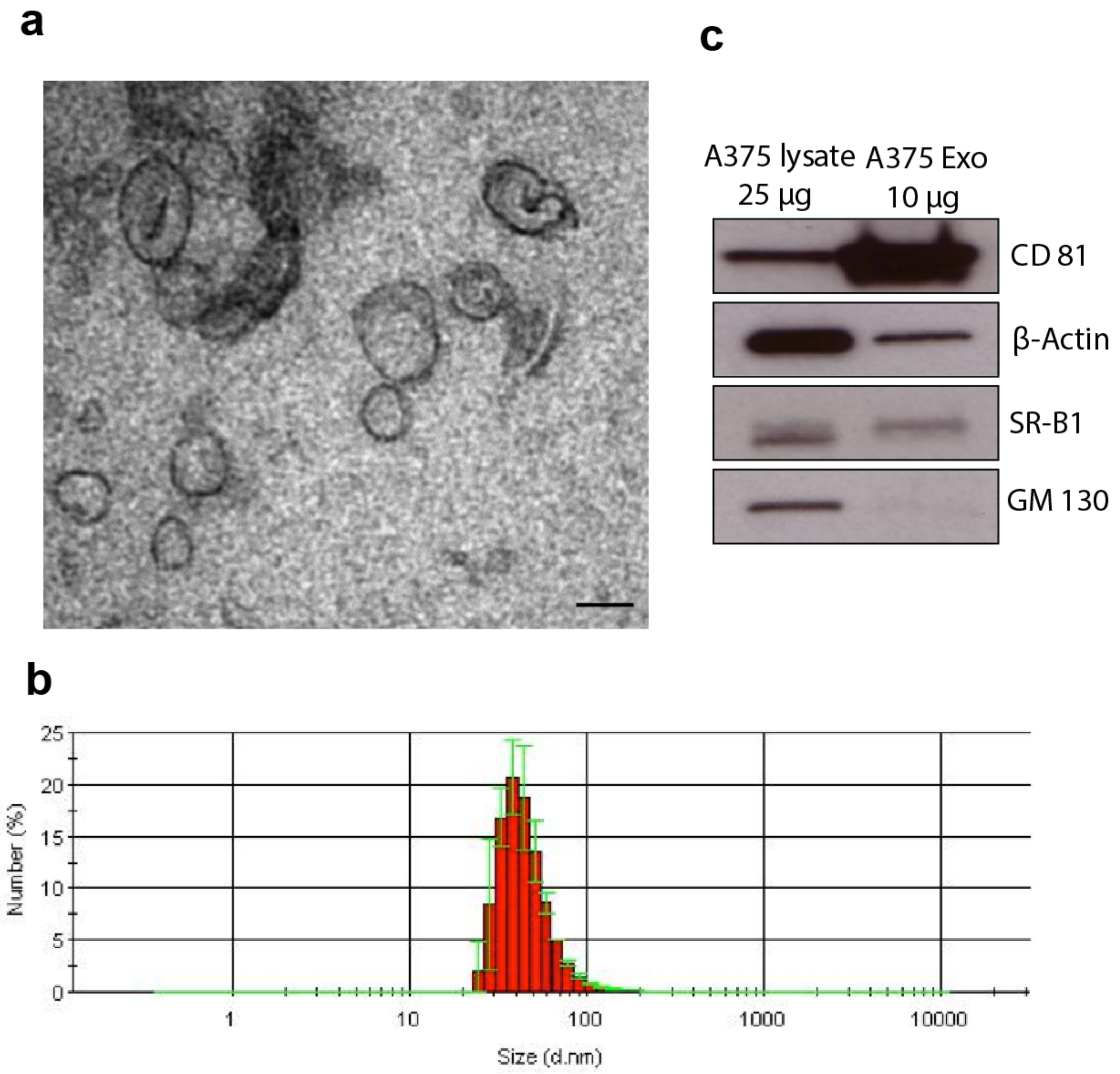

Supplementary Figure 1: Characterization of A375 melanoma exosomes: size, morphology and molecular markers: (**a**) Transmission electron micrograph (TEM) of A375 exosomes isolated by differential ultracentrifugation. The isolated exosomes display typical cup-shaped morphology (scale bar = 50 nm). (**b**) Dynamic light scattering in shows the expected exosome size (20-100 nm hydrodynamic diameter). (**c**) Western blot shows the enrichment of CD81, a member of the tetraspanin family and exosomal marker, in exosome fraction. Additionally, western blotting reveals the absence of the Golgi marker protein 130 (GM 130) demonstrating that prepared exosomes are devoid of components of cellular organelles. Finally, the western blot shows SR-B1 presence in the A375 cell lysate and in exosomes.

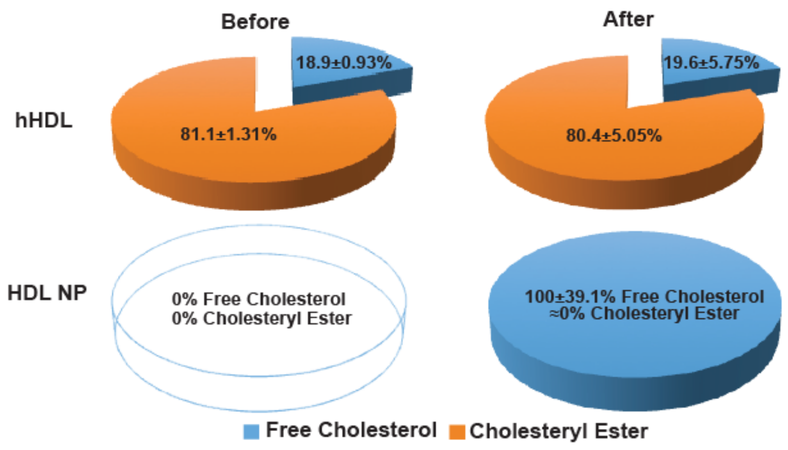

Supplementary Figure 2: HDL NPs efflux free cholesterol from melanoma cells: Pie charts show the content, percent of total measured cholesterol, of free cholesterol and cholesteryl ester to hHDL and HDL NPs **before (left)** and **after (right)** cholesterol efflux assay in A375 melanoma cells.

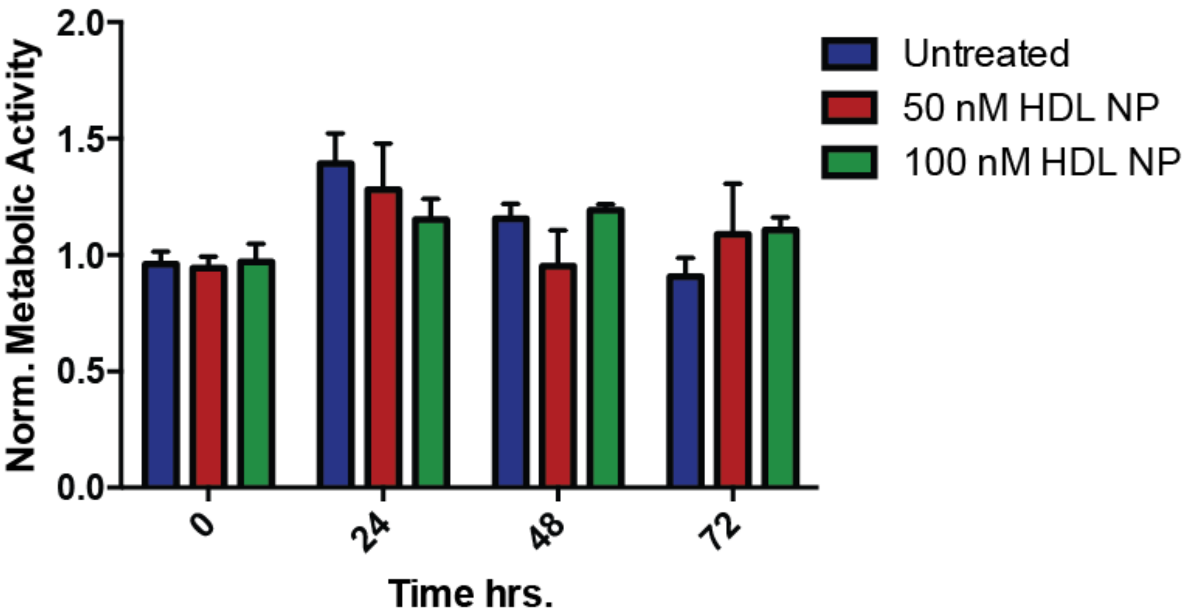

Supplementary figure 3: HDL NPs have no effect on cellular viability. A375 melanoma cells were treated with 50 and 100 nM HDL NP and the cytotoxicity was measured using 3-(4,5-dimethylthiazol-2-yl)-5-(3-carboxymethoxyphenyl)-2-(4-sulfophenyl)-2H-tetrazolium (MTS) assay at T = 0, 24, 48, and 72 hours after HDL NP treatment.

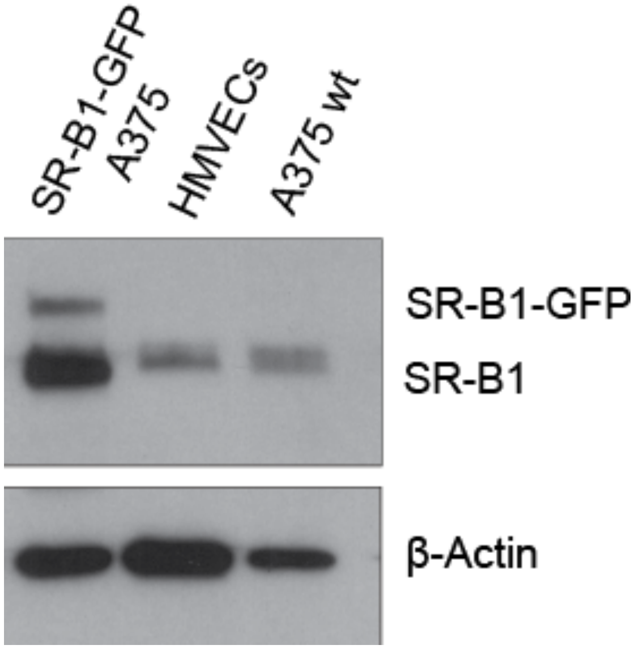

Supplementary Figure 4: Expression of SR-B1 and GFP-SR-B1 in A375 cells and HMVECs. Western blot shows for SR-B1 in both A375 cells and HMVECs using an anti-SR-B1 monoclonal antibody. Note: GFP-SR-B1 fusion protein, characterized by increased molecular weight in A375 cells transfected with appropriate construct (lane 1).

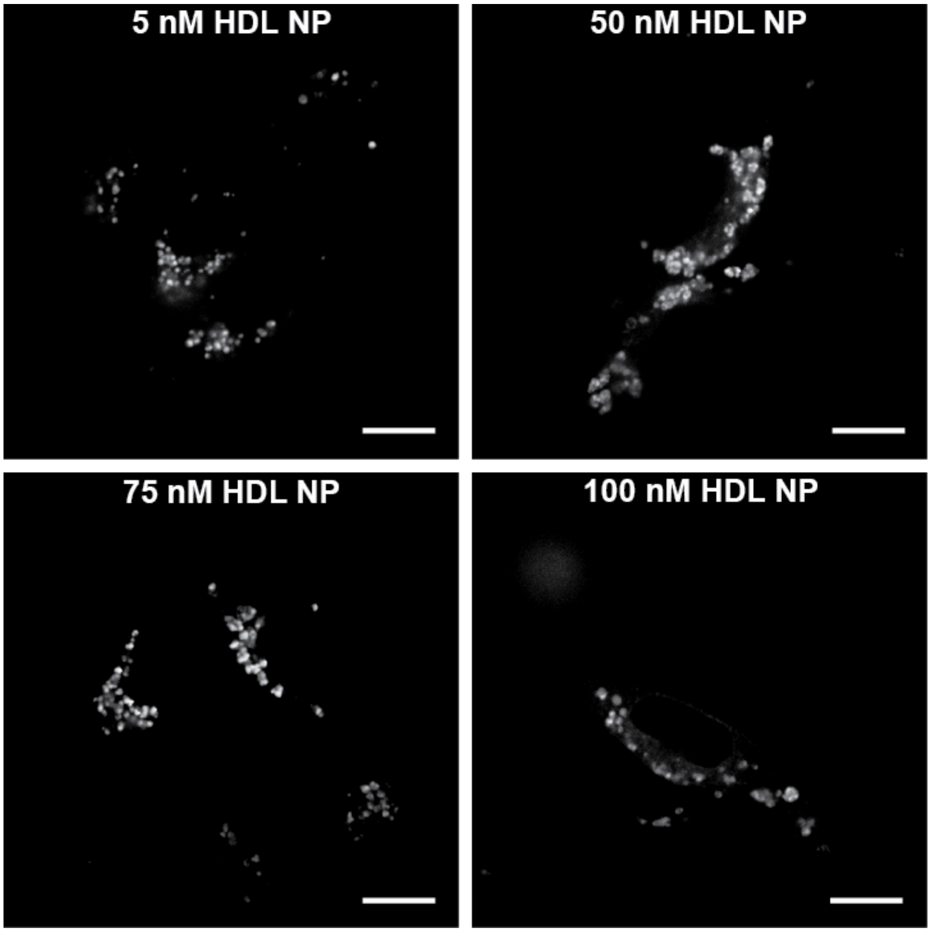

Supplementary Figure 5: Clustering of SR-B1 is dependent on HDL NP dose. Time lapse images of GFP-SR-B1 expressing A375 melanoma cells after treatment with 5 nM, 50 nM 75 nM and 100 nM doses of HDL NP. Representative images were acquired after 24 hrs HDL NP treatment.

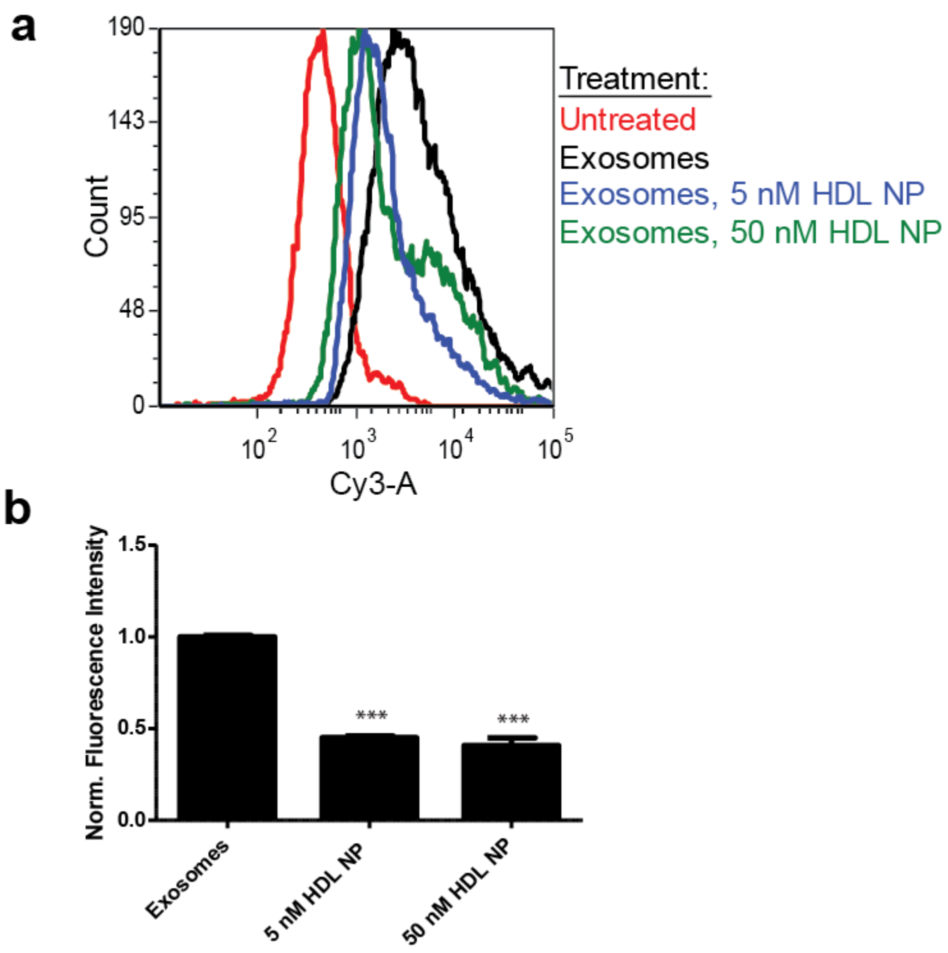

Supplementary Figure 6: HDL NP treatment inhibits exosome uptake in A375 melanoma cells expressing GFP-SR-B1. A375 cells expressing GFP-SR-B1 were treated with exosomes in the presence of 0, 5, and 50 nM HDL NPs. As was observed for the wild-type (untransfected) A375 cells, exosomes and HDL NP treatment reduces exosome uptake A375 cells expressing GFP-SR-B1. Graph **b** shows the average fluorescence intensity of cells analyzed by flow cytometry in **a**. (*** represents P<0.001 as compared to exosome only condition).

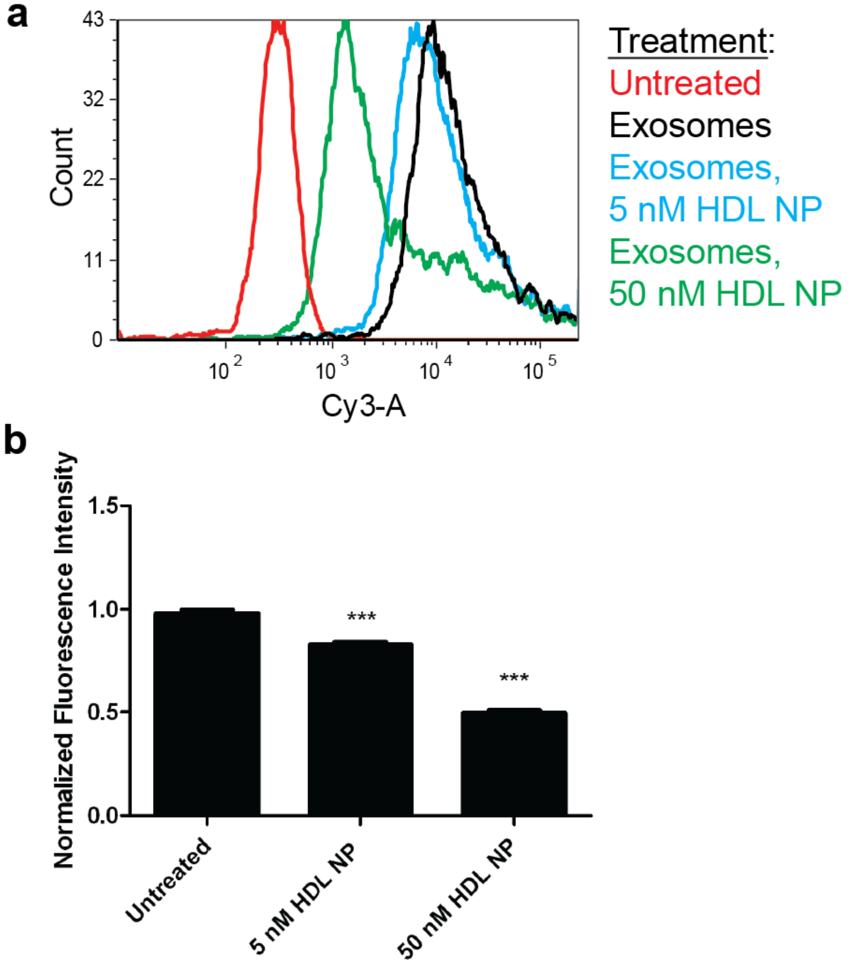

Supplementray Figure 7: The inhibition of exosome uptake after treatment with HDL NP is not due to extracellular interaction of exosomes and HDL NP. A375 melanoma cells were pre-treated for 12 hours with HDL NP 5 and 50 nM. Excess HDL NPs were then washed 2 times in PBS (**a**) Flow cytometry analysis of exosome uptake after A375 cell pre-treatment with HDL NP. Graph **b** shows the average fluorescence intensity of cells analyzed by flow cytometry in **a**. (*** Represents P<0.001 as compared to exosome only condition).

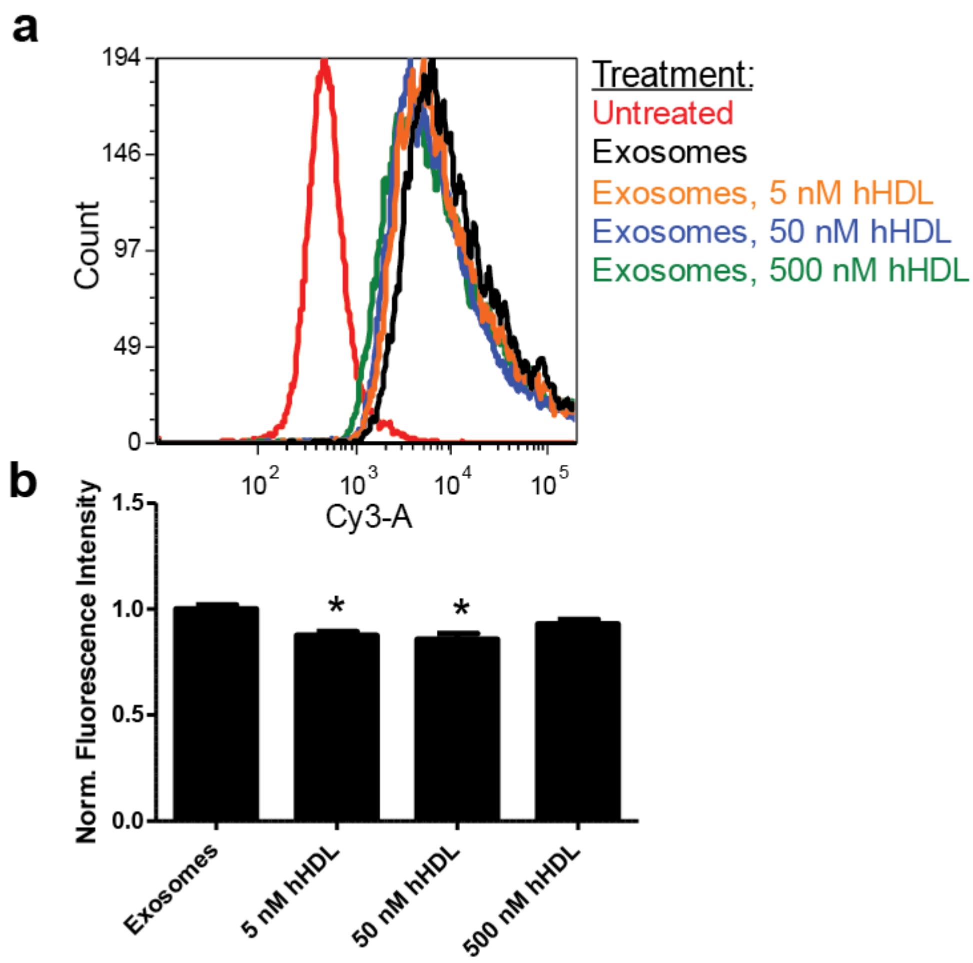

Supplementary Figure 8: hHDL has only a modest effect on exosome uptake by A375 cells. (**a**) Exosomes were labeled using DiI and their uptake by A375 cells in the presence of 0, 5, 50, 500 nM hHDL was measured using flow cytometry. In contrast to HDL NP treatment, there the reduction in exosome uptake does not exceed 15%, even at 500 nM hHDL. Graph **b** shows the average fluorescence intensity of cells analyzed by flow cytometry in **a**. (* Represents $P<0.05$ as compared to exosome only condition)

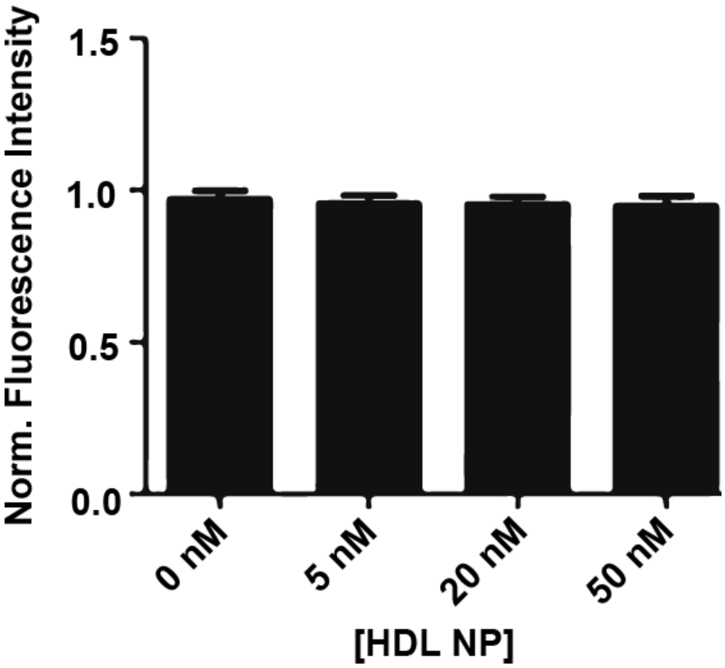

Supplementary Figure 9: HDL NP displays no fluorescence quenching when incubated when DiI labeled exosomes. DiI labeled exosomes were incubated with HDL NP at concentrations of: 0, 5, 20, 50 for 6 hours and read on a Biotek Synergy plate-reader. Co-incubation of HDL NP with exosomes did not reduce the normalized fluorescence intensity as compared to untreated control samples.

Supplementary Video 1. Time-lapse confocal fluorescence microscopy of GFP-SR-B1 imaged every 2 seconds over the course of 2 minutes. The video shows the natural dynamics of SR-B1 in lipid rafts over time.

Supplementary Video 2. Time-lapse confocal fluorescence microscopy of GFP-SR-B1 imaged every 2 seconds over the course of 2 minutes after treatment with 30 nM HDL NP for 24 hours. The video shows clustering and decreased movement of SR-B1.

Supplementary Video 3. The SR-B1-GFP domains in **Supplementary Video 1** were segmented using a semi-automated approach (Materials and Methods section) in order to quantify GFP-SR-B1 size, intensity and dynamics.

Supplementary Video 4. The SR-B1-GFP domains in **Supplementary Video 2** were segmented using a semi-

automated approach (Materials and Methods section) in order to quantify GFP-SR-B1 size, intensity and dynamics.

Supplementary Video 5. Time-lapse confocal fluorescence microscopy of GFP-SR-B1 imaged every 2 seconds over the course of 2 minutes after treatment with melanoma cell-derived exosomes for 24 hours. There was no change in the dynamics of SR-B1 after treatment with exosomes.
